# Bilateral Congenital Stromal Corneal Cysts: Report of a Unique Case

**DOI:** 10.7759/cureus.41446

**Published:** 2023-07-06

**Authors:** Khalid B Alburayk, Saleh S Alghamdi, Musab A Alsubaie, Wejdan S Alghamdi, Fatimah Alzaher, Abdulaziz I Alsomali

**Affiliations:** 1 Department of Ophthalmology, King Fahd Hospital of the University, Imam Abdulrahman Al Faisal University, Dammam, SAU; 2 Department of Ophthalmology, College of Medicine, Imam Abdulrahman Bin Faisal University, Dammam, SAU; 3 Department of Ophthalmology, King Fahad Military Medical Complex, Dhahran, SAU; 4 Department of Ophthalmology, Albaha University, Albaha, SAU; 5 Department of Ophthalmology, Dhahran Eye Specialist Hospital, Dhahran, SAU; 6 Department of Ophthalmology, King Faisal University, Al-Hasa, SAU

**Keywords:** keratoplasty, ultrasound biomicroscopy, intrastromal cyst, corneal opacity, congenital rubella

## Abstract

The purpose of this report is to provide a comprehensive account of an exceptional case involving the presentation of congenital rubella syndrome (CRS) in a newborn. Furthermore, it aims to document the successful regression of CRS through medical treatment alone. We present the case of a five-day-old infant who was referred to our facility as a CRS case. The patient presented with bilateral white corneal opacity, which was observed shortly after birth. The mother was diagnosed as rubella-positive during pregnancy. Upon the initial examination under anesthesia, both eyes exhibited central white corneal opacity accompanied by large intrastromal cysts. Although a few breaks in Descemet's membrane were observed in both eyes, there were no signs of vascularization or the presence of iridocorneal or lenticular-corneal adhesions. After undergoing medical treatment consisting of topical sodium chloride and steroids, the cysts in both eyes completely regressed. Subsequently, the patient underwent penetrating keratoplasty to further address the dense scar. This case enhances our comprehension of ophthalmological complications associated with CRS and provides valuable insights into alternative therapeutic approaches for corneal stromal cysts.

## Introduction

Congenital rubella syndrome (CRS) is a prevalent congenital infection known for its wide range of severe ophthalmic and systemic complications [1]. It typically emerges during the first trimester of pregnancy in women who have experienced rubella, carrying a risk as high as 81% [2]. The incidence of CRS has significantly declined in developed countries due to the implementation of rubella vaccination. However, in developing countries like India, CRS remains a significant health concern [2]. In 2010, the estimated incidence of CRS was reported to be between 90 and 121 cases per 100,000 live births [3]. The national immunization program in India included rubella vaccination in 2017 and 2018, achieving a national coverage rate of 87.33% during that period [3]. The association between maternal rubella infection during pregnancy, congenital cataracts, and hearing impairment was initially identified by Gregg in 1941. This discovery shifted the clinical understanding of rubella from being primarily a mild infection in children to a serious disease in adult women with teratogenic effects [4]. Ocular anomalies, including glaucoma, cataracts, microphthalmia, and pigmentary retinopathy, are defining characteristics of CRS [5]. The objective of our study was to present a case of CRS featuring a corneal stromal cyst and review the documented ophthalmological manifestations associated with CRS.

## Case presentation

**Figure 1:**
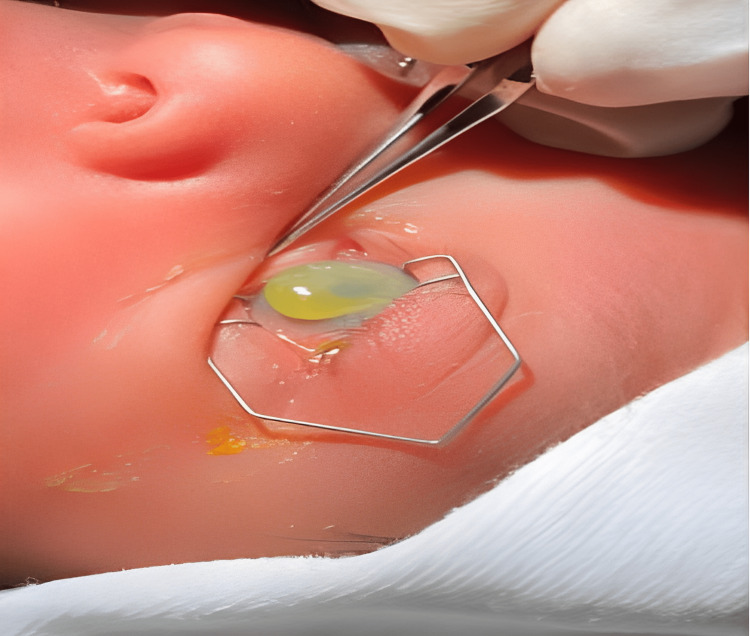
Left eye photo at the initial presentation revealing a noticeable large bulging cyst.

**Figure 2:**
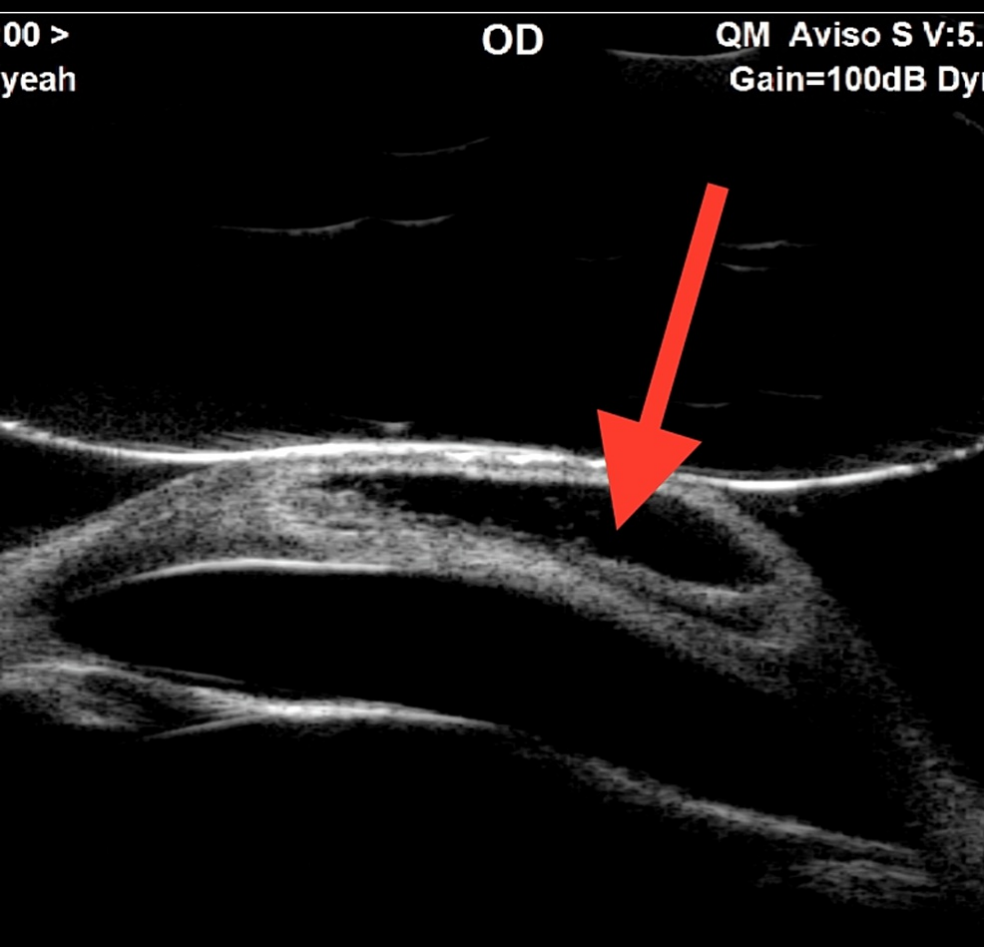
UBM of the right eye showing an intrastromal large fluid-filled cyst. UBM, ultrasound biomicroscopy

**Figure 3:**
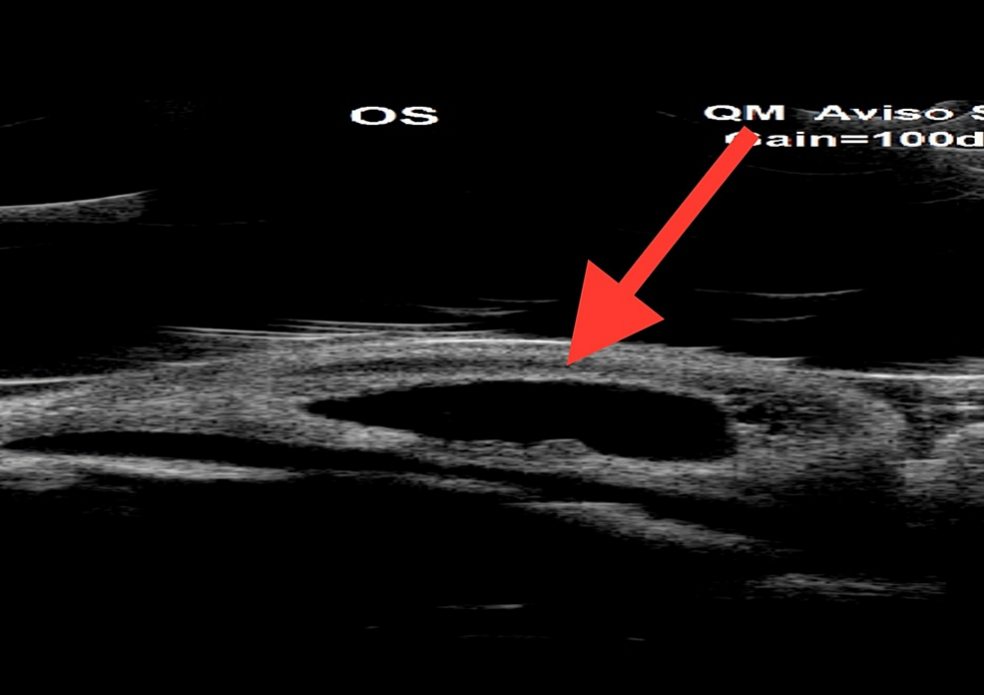
UBM of the left eye showing an intrastromal large fluid-filled cyst. UBM, ultrasound biomicroscopy

**Figure 4:**
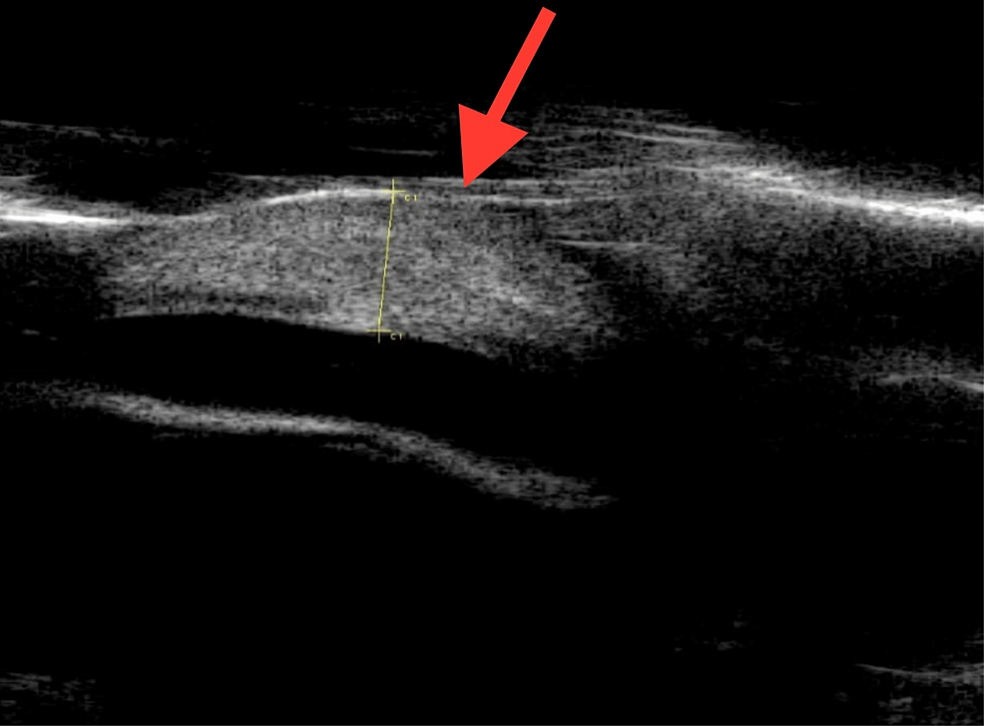
UBM of the left eye one month after medical treatment showing complete resolution of the cyst (the right eye also showed a similar finding). UBM, ultrasound biomicroscopy

**Figure 5:**
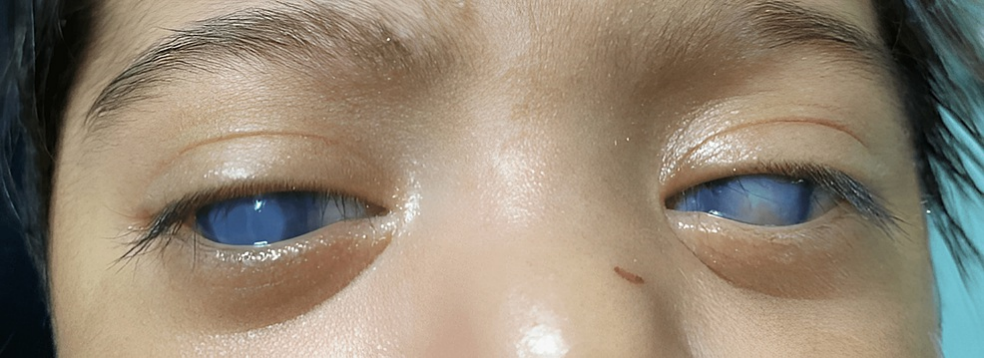
Both eyes showing bilateral large central corneal scars with new stromal neovascularization.

A five-day-old male infant with a confirmed diagnosis of CRS was referred to our facility for examination due to the presence of white corneal opacification in both eyes. The patient's mother also had a confirmed diagnosis of reactive rubella during pregnancy. Upon the initial examination under anesthesia, both eyes exhibited central white corneal opacity along with large intrastromal cysts. Although a few breaks in the Descemet membrane were identified in both eyes, no signs of neovascularization or iridocorneal or lenticular-corneal adhesions were observed (Figure 1). Ultrasound biomicroscopy (UBM) revealed the presence of large bilateral intrastromal corneal cysts without any sign of iridocorneal or lenticular-corneal adhesions (Figures 2-3). B-scan ultrasonography (USG) confirmed the normal condition of the posterior segment in both eyes. Following medical treatment involving topical sodium chloride and steroids, the patient demonstrated gradual improvement in both cysts. Approximately one month later, complete regression of both cysts was observed, as evidenced by UBM (Figure 4). However, this regression resulted in bilateral large central corneal scars accompanied by new stromal vascularization (Figure 5). Subsequently, the patient underwent penetrating keratoplasty (PKP) at the age of five months to address the persistent corneal scars in an otherwise normal globe. Histopathological examination of the cornea confirmed the presence of scars in the otherwise intact corneal layers.

## Discussion

CRS is a prevalent congenital infection characterized by various clinical manifestations, including heart defects, hearing impairments, and ophthalmological abnormalities [6]. The most commonly observed and extensively documented ophthalmic associations include cataracts, pigmentary retinopathy, microphthalmia, and glaucoma. However, this case report presented a unique ophthalmic finding of stromal corneal cysts in association with CRS, which, to the best of our knowledge, has not been previously reported in the existing literature. Corneal cysts are fluid-filled lesions that can occur on or within the layers of the cornea. They can be classified into two categories: stromal cysts and epithelial cysts. Epithelial cysts, resembling small dome-shaped lesions filled with clear fluid, develop in the outer layer of the cornea due to trauma or breakdown [7]. They may or may not cause visual impairment and can occur at any age. On the other hand, stromal cysts, although rare, can develop in the middle layer of the cornea due to trauma or developmental issues. These cysts are larger and filled with thicker fluid or semisolid substances, and depending on their size and location, they can affect vision. While previous studies have reported cases of corneal cysts, they have not specifically addressed their association with CRS. Furthermore, all reported cases of corneal stromal cysts have been treated surgically, regardless of the underlying cause [8-10]. The rationale behind the use of topical steroids is their potential effect on enhancing the pump function of the endothelium, which may cause shrinkage and hydration of the fluid within the cyst. While hypertonic agents are intended to be used to make the tear film more tonic and subsequently draw the fluid osmotically from the cornea into the tears, they not only have a maximum effect on epithelial edema but also have a beneficial effect on stromal edema [11-16]. Surgical procedures such as excision and curettage, puncture and aspiration, lamellar keratoplasty, and PKP have been utilized to treat corneal cysts. Puncture and aspiration involve collapsing the cyst by removing its contents, while excision and curettage require surgical removal of the cyst. PKP involves replacing the entire cornea, while lamellar keratoplasty replaces only the affected layers of the cornea. The choice of procedure depends on various factors, including the type, location, size, and health of the patient's eyes. To the best of our knowledge, this is the first reported case of a corneal stromal cyst in the context of congenital rubella, demonstrating a positive response to medical management without the need for surgical intervention. For the management of ophthalmic complications associated with CRS, specific antiviral treatments are not available, and management primarily focuses on providing supportive care and addressing associated complications. Medical management aims to control complications such as ocular inflammation and elevated intraocular pressure. Surgical interventions, such as cataract extraction and glaucoma surgery, may be necessary to address specific ophthalmic abnormalities [17]. In this case of corneal cysts, we chose a medical management approach that involved the use of topical sodium chloride, topical steroids, and lubricants. This conservative management resulted in a favorable outcome, with complete resolution of the cysts in both corneas. However, due to subsequent visually significant scarring in both corneas and the need to eliminate the risk of amblyopia, subsequent PKP was performed on both eyes with fine-needle diathermy to address the corneal vascularization. This case not only introduces a novel ophthalmological finding in CRS but also contributes to expanding our understanding of this syndrome. Moreover, the successful medical management of corneal stromal cysts opens new avenues for managing this condition.

## Conclusions

In conclusion, this case study brings attention to the infrequent presentation of corneal stromal cysts in a newborn with CRS, emphasizing the importance of identifying uncommon ophthalmological manifestations associated with CRS. The effective nonsurgical management of corneal stromal cysts demonstrates a noninvasive treatment option. This case contributes to a broader understanding of ophthalmological complications and provides valuable insights into alternative therapeutic approaches for corneal stromal cysts.
